# Effects of Ginsenoside Rb1 on the Crosstalk between Intestinal Stem Cells and Microbiota in a Simulated Weightlessness Mouse Model

**DOI:** 10.3390/ijms25168769

**Published:** 2024-08-12

**Authors:** Beibei Zong, Jingyi Wang, Kai Wang, Jie Hao, Jing-Yan Han, Rong Jin, Qing Ge

**Affiliations:** 1 Department of Integration of Chinese and Western Medicine, School of Basic Medical Sciences, Peking University, Beijing 100191, China; zongbeibei@pku.org.cn (B.Z.); hanjingyan@bjmu.edu.cn (J.-Y.H.); 2 Department of Immunology, School of Basic Medical Sciences, Peking University, Beijing 100191, China; 1810305326@pku.edu.cn (J.W.); wangkai@bjmu.edu.cn (K.W.); haojie@bjmu.edu.cn (J.H.); 3 NHC Key Laboratory of Medical Immunology, Peking University, Beijing 100191, China

**Keywords:** intestinal stem cells, microbiota, simulated microgravity, ginsenoside Rb1

## Abstract

Exposure to the space microenvironment has been found to disrupt the homeostasis of intestinal epithelial cells and alter the composition of the microbiota. To investigate this in more detail and to examine the impact of ginsenoside Rb1, we utilized a mouse model of hindlimb unloading (HU) for four weeks to simulate the effects of microgravity. Our findings revealed that HU mice had ileum epithelial injury with a decrease in the number of intestinal stem cells (ISCs) and the level of cell proliferation. The niche functions for ISCs were also impaired in HU mice, including a reduction in Paneth cells and Wnt signaling, along with an increase in oxidative stress. The administration of Rb1 during the entire duration of HU alleviated the observed intestinal defects, suggesting its beneficial influence on epithelial cell homeostasis. Hindlimb unloading also resulted in gut dysbiosis. The supplementation of Rb1 in the HU mice or the addition of Rb1 derivative compound K in bacterial culture in vitro promoted the growth of beneficial probiotic species such as *Akkermansia*. The co-housing experiment further showed that Rb1 treatment in ground control mice alone could alleviate the defects in HU mice that were co-housed with Rb1-treated ground mice. Together, these results underscore a close relationship between dysbiosis and impaired ISC functions in the HU mouse model. It also highlights the beneficial effects of Rb1 in mitigating HU-induced epithelial injury by promoting the expansion of intestinal probiotics. These animal-based insights provide valuable knowledge for the development of improved approaches to maintaining ISC homeostasis in astronauts.

## 1. Introduction

Intestinal stem cells (ISCs) are crucial for maintaining the balance and renewal of the intestinal epithelium [1]. These cells generate daughter cells that enter the transit-amplifying (TA) compartment, where they undergo proliferation and differentiation into mature intestinal epithelial cells, including absorptive cells and secretory cells [2]. The regulation of ISC proliferation and function relies on a supportive microenvironment involving various cell types, such as granular Paneth cells, intestinal subepithelial myofibroblasts, stromal cells, and immune cells [3]. Additionally, multiple signaling pathways, including Wnt, Notch, BMP, and EGF, contribute to ISC regulation [4]. 

The intestinal microbiota plays a critical role in development, health, and overall performance. It influences the breakdown of complex nutritional components, the production of bioactive metabolites, the development of the immune system, and the defense against colonization by harmful microbes. The gut microbiota can directly or indirectly regulate ISCs. This regulation involves pattern recognition receptors (PRRs), as well as the redox state and oxygen concentration in the intestine [5,6,7]. Microbiota-derived metabolites, such as short-chain fatty acids and secondary bile acids, also impact ISC activity [8,9]. 

Exposure to microgravity leads to intestinal epithelial injury and dysbiosis. Studies on rats flown on COSMOS and Foton M3 missions and the hindlimb unloaded (HU) mouse model, which simulates microgravity conditions by exhibiting cephalic fluid shift, repositioning of viscera, and musculoskeletal unloading, have shown reduced villi length, decreased crypt depth, and impaired mucin production in the intestinal epithelium [10,11,12]. Furthermore, HU models have demonstrated reduced intestinal epithelial proliferation, increased epithelial apoptosis and permeability, and susceptibility to bacterial infection [13,14,15,16]. These conditions also disrupt the gut microflora composition [14,15,17,18,19,20]. A decrease in beneficial species, such as *Akkermansia* and *Lactobacillus*, and an increase in harmful ones, such as *Lachnospiraceae*, were shown in a three-week HU experiment [20,21]. Administering certain bacterial-conditioned media or feces from ground mice to HU mice can partially improve epithelial integrity and other abnormalities, suggesting the involvement of dysbiosis in HU-induced intestinal epithelial injury [14,15,22]. However, the impact of dysbiosis on ISCs in HU mice remains unknown. 

Ginsenosides, triterpenoid saponins found in *Panax ginseng* Meyer, possess energizing properties and can strengthen the immune system [23]. Ginsenoside Rb1 is a prominent component of total ginsenosides and has demonstrated various beneficial effects on intestinal health [24]. Rb1 can alleviate colitis in mice by mitigating endoplasmic reticulum (ER) stress through the activation of the Hrd1 signaling pathway, reduce ischemia/reperfusion-induced intestinal injury by activating the PI3K/AKT/Nrf2 pathway [25], improve intestinal aging by regulating the expression of sirtuins [26], promote intestinal epithelial wound healing in rats by activating ERK and Rho signaling [27], and protect against intestinal mucosa damage caused by peritoneal air exposure [28]. However, it is currently unknown whether Rb1 can improve intestinal homeostasis in HU mice.

To address this, we conducted a 28-day HU mouse model study to investigate the potential positive impact of Rb1 on ISCs and the epithelial barrier. We also explored the role of the microbiota in modulating the effects of Rb1 on the intestinal epithelium. 

## 2. Results

### 2.1. Rb1 Enhanced Intestinal Barrier Function in HU Mice

To investigate the impact of Rb1 on the intestinal epithelium in HU mice, we conducted a study using female C57BL/6 mice that were hindlimb unloaded for 4 weeks. Rb1, dissolved in water at a concentration of 50 mg/kg, was administered to the mice via oral gavage starting 3 days before the initiation of the hindlimb unloading (HU-Rb1). Control groups consisted of mice on the ground, treated with either water (Ctrl-H_2_O) or the same dosage of Rb1 (Ctrl-Rb1), and HU mice, treated with water (HU-H_2_O). 

**Figure 1:**
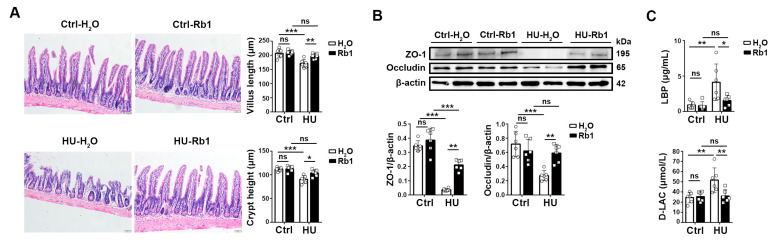
Rb1 treatment alleviated HU-induced intestinal injury in mice. Female C57BL/6 mice at 8–10 weeks of age were hindlimb unloaded (HU) for a duration of 4 weeks. Rb1 was dissolved in water at a concentration of 50 mg/kg and was administered daily to the mice via oral gavage starting 3 days before the initiation of the hindlimb unloading (HU-Rb1). Control groups consisted of mice on the ground, treated with either the same amount of water (Ctrl-H_2_O) or the same dosage of Rb1 (Ctrl-Rb1), and HU mice, treated with water (HU-H_2_O). *n* = 6. (**A**) H&E staining of the ileum epithelium. The scale bar is 20 μm. The representative sections are shown on the left. Villus length and crypt depth are summarized on the right. (**B**) Western blotting of ZO-1 and Occludin expressions in ileum epithelium. The data are representative of 3 independent experiments. Gray values of ZO-1 and Occludin were determined for statistical analysis at the bottom. *n* = 6. (**C**) Comparison of plasma LPS-binding protein (LBP) and D-lactate (D-LAC) in mice. Data are shown as mean ± SD. * *p* < 0.05, ** *p* < 0.01, *** *p* < 0.005, ns, non-significant by unpaired two-tailed Student’s *t*-test.

Hematoxylin and eosin (H&E) staining was performed on ileum sections obtained from both ground control mice and HU mice. A significant reduction in villi length and crypt depth was observed in the HU-H_2_O mice compared to the ground control mice (Ctrl-H_2_O and Ctrl-Rb1, Figure 1A). Increased cell death and shedding were observed in the HU-H_2_O samples. Immunoblot analysis revealed reduced protein levels of Occludin and ZO-1, which are essential molecules involved in tight junctions, in the HU ileum (Figure 1B). However, Rb1 treatment mitigated these epithelial defects in the HU mice (HU-Rb1, Figure 1A,B). No notable differences were observed between the ground control mice with and without Rb1 treatment. 

Furthermore, the HU mice exhibited significantly higher levels of serum LPS-binding protein (LBP) and D-lactate (D-LAC), indicating impaired mucosal barrier function (Figure 1C). Rb1 treatment decreased LBP and D-Lac in the HU mice, reaching similar levels as the ground control mice. These results suggest that Rb1 protects the HU mice from intestinal injury. 

### 2.2. Rb1 Promoted ISC Homeostasis in HU Mice

**Figure 2:**
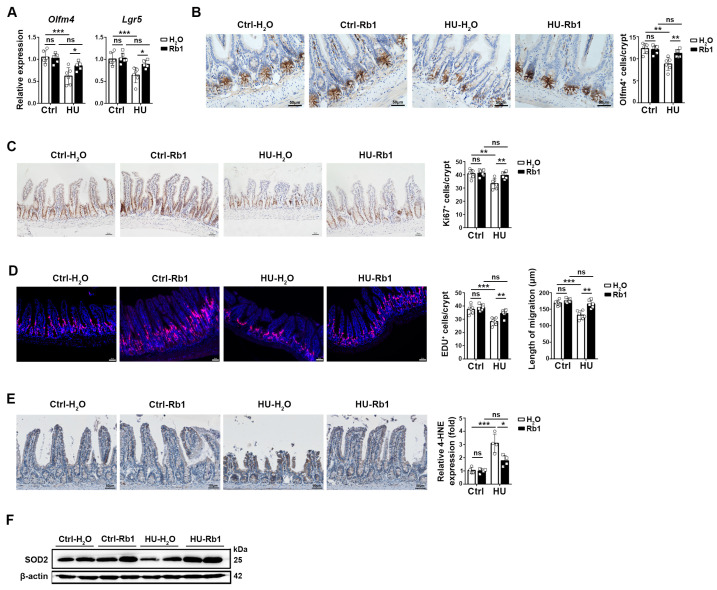
Rb1 treatment promoted ISC homeostasis in the ileum of HU mice. (**A**) Quantitative PCR analysis of *Olfm4* and *Lgr5* transcription in ileum epithelial cells. *n* = 6. (**B**) Comparison of immunostaining of ileum sections with Olfm4 antibody. The representative sections are shown on the left. The scale bar is 50 μm. The numbers of Olfm4-positive cells per crypt are summarized on the right. In total, 20–30 crypts were counted. *n* = 6. (**C**) Comparison of Ki67 staining in ileum sections. The representative sections are shown on the left. The scale bar is 50 μm. The quantification of Ki67-positive cells per crypt is summarized on the right. In total, 20–30 crypts of each mouse were counted. (**D**) Comparison of epithelial cell proliferation and migration with EDU-specific immunostaining. *n* = 6. HU and Ctrl mice with and without Rb1 were given EDU (5-Ethynyl-2′-deoxyuridine) for 12 h before harvest, and the ileum sections were stained with anti-EDU antibody. The representative sections are shown on the left panel. The scale bar is 50 μm. The quantification of EDU-positive cells per crypt is summarized on the right panel. In total, 20–30 crypts of each mouse were counted. The length from the crypt base to which EDU-positive cells migrated the farthest was defined as the epithelial migration distance. At least 20 crypts of each mouse were calculated to represent the migration distance, as shown on the right. (**E**) Comparison of immunostaining of ileum sections with a specific antibody against 4-hydroxynonenal (4-HNE), a lipid peroxidation product that indicates oxidative stress. The representative sections are shown on the left. The scale bar is 50 μm. The numbers of 4-HNE-positive cells per crypt are summarized on the right. In total, 20–30 crypts were counted. *n* = 4. Data are shown as mean ± SD. * *p* < 0.05, ** *p* < 0.01, *** *p* < 0.005, ns, non-significant by unpaired two-tailed Student’s *t*-test. (**F**) Western blotting of the expression of mitochondrial superoxide dismutase 2 (SOD2) in the ileum epithelium. The data are representative of 2 independent experiments.

We next investigated whether the homeostasis of ISCs in the HU ileum was affected and if Rb1 could alleviate these changes. In comparison to the ground control mice, the HU ileum exhibited reduced expression of *Lgr5* and *Olfm4* in the ileum epithelial cells, along with a decreased number of Olfm4^+^ cells per crypt (Figure 2A,B). HU mice treated with Rb1 showed elevated *Olfm4* and *Lgr5* expression and an increased number of Olfm4^+^ cells, reaching levels comparable to the ground control micet (Figure 2A,B).

To further examine the impact of HU on epithelial cell proliferation, we assessed the levels of Ki67 staining and EDU incorporation. As depicted in Figure 2C,D, HU mice displayed reduced levels of epithelial cell proliferation, as indicated by Ki67 or EDU staining. However, Rb1 treatment in HU mice alleviated this decrease in proliferation. Notably, the migration distance of EDU^+^ epithelial cells was also increased in Rb1-treated HU mice compared to HU mice without Rb1 (Figure 2D). 

Oxidative stress plays a significant role in regulating ISC proliferation and development. In comparison to the ileum epithelial cells of ground control mice, those of HU mice exhibited increased levels of oxidative stress, as evidenced by higher n-6 fatty acid oxidation (4-hydroxy-2-nonenal (4-HNE)-positive cells) and decreased levels of the antioxidant molecule SOD2 (Figure 2E,F). These results suggest that Rb1 treatment mitigates epithelial oxidative stress in HU mice (Figure 2E,F). 

### 2.3. Rb1 Improved the Niche Function of ISCs

**Figure 3:**
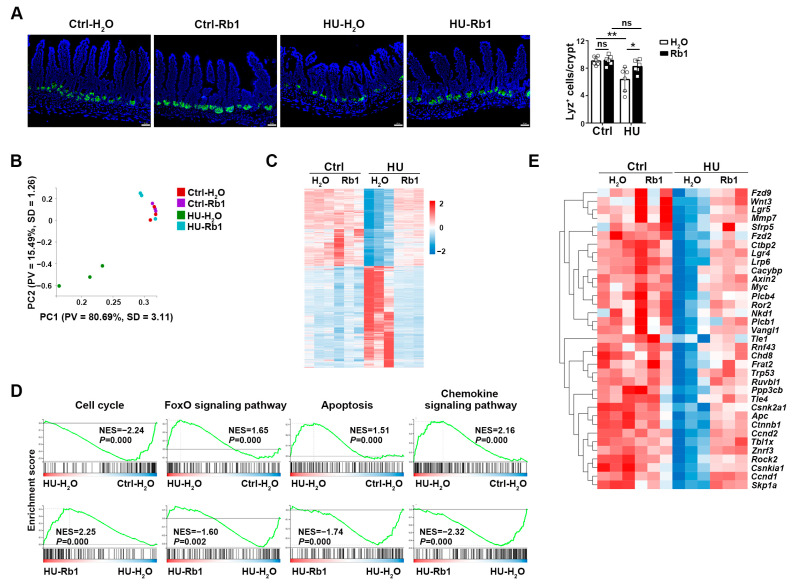
Rb1 treatment improved the niche function of ISCs. (**A**) Comparison of immunostaining of ileum sections with Lysozyme (Lyz) antibody. The representative sections are shown on the left. The scale bar is 50 μm. The numbers of Lysozyme-positive cells per crypt are summarized on the right. In total, 20–30 crypts were counted. *n* = 6. Data are shown as mean ± SD. * *p* < 0.05, ** *p* < 0.01, ns, non-significant by unpaired two-tailed Student’s *t*-test. (**B**) Principal component analysis (PCA) of ileum epithelial cells in HU and Ctrl mice (*n* = 3). The ileum epithelial cells were purified from 4-week HU or ground control mice and were subjected to RNA sequencing. (**C**) Heatmap of differentially expressed genes among various groups. (**D**) Gene enrichment analysis by GSEA of the transcriptome profiles derived from each group. NES, normalized enrichment score. (**E**) Heatmap of genes enriched in the Wnt signaling pathway.

The function of the niche is crucial for maintaining ISC homeostasis and proliferation. To investigate whether Rb1 could improve niche function, we first compared Paneth cells using lysozyme staining. As depicted in Figure 3A, the numbers of Lyz^+^ Paneth cells in the crypt were significantly reduced in the HU ileum but increased in the Rb1-treated HU ileum.

We then examined the transcriptome of ileum epithelial cells in these mice. Hierarchical clustering and a heatmap of differentially expressed genes revealed that samples from Rb1-treated HU mice clustered together with ground control mice while being distinct from HU mice without Rb1 treatment (Figure 3B,C). Gene Ontology and the Molecular Signature Database (GSEA) analysis demonstrated that genes related to the cell cycle were downregulated, while those related to the FoxO signaling pathway, apoptosis, and chemokine signaling pathway were upregulated in HU samples without Rb1 (Figure 3D). However, Rb1 treatment alleviated these changes in HU mice (Figure 3D). Importantly, a set of genes involved in the Wnt signaling pathway (Figure 3E), which is essential for ISC maintenance, exhibited a significant reduction in HU mice but were restored in Rb1-treated mice (Figure 3E). Collectively, these results suggest that Rb1 treatment protects ISCs and their niche from HU-induced injury. 

### 2.4. Rb1 Attenuated Gut Dysbiosis in HU Mice

**Figure 4:**
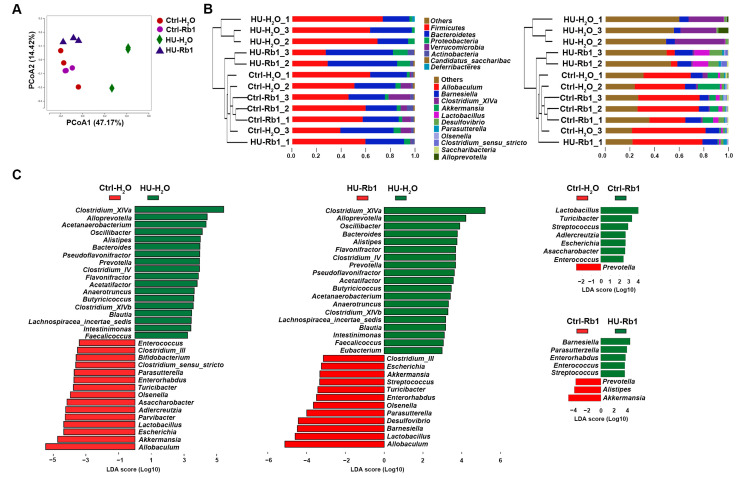
Rb1 attenuated gut dysbiosis in HU mice. (**A**) Principal co-ordinates analysis (PCoA) of the cecum contents of HU-H_2_O, HU-Rb1, Ctrl-H_2_O, and Ctrl-Rb1 mice by 16S microbial sequencing. *n* = 3. (**B**) Commensal diversity analysis of mice at phyla (left) and genera (right) levels. The clustering of microbiota communities was determined by unweighted UniFrac analysis. (**C**) Histogram of unique biomarker bacteria in indicated groups. The LDA effect size (>2-fold) was used to detect unique biomarkers.

To assess the impact of Rb1 on the intestinal microbiota, we conducted 16S rRNA sequencing of cecum contents from the mice. The phylogenetic differences within the gut microbiome were assessed by Principal Co-ordinates Analysis (PCoA). As illustrated in Figure 4A, the microbiota of Rb1-treated HU samples clustered together with the ground control samples, while HU mice without Rb1 treatment exhibited a distinct profile from the other groups. We further analyzed the relative abundance of the predominant bacterial clades. At the phylum level, Firmicutes and Bacteroidetes were the dominant microflora in all groups, with a slight decrease in Firmicutes and an increase in Bacteroidetes in the ground control groups and the Rb1-treated HU group (Figure 4B). At the genus level, *Clostridium_XIVa* was the dominant microflora in the HU-H_2_O group, while *Allobaculum* and *Barnesiella* were dominant in other groups, including the HU-Rb1 group (Figure 4B). Discriminant analysis effect size (LEfSe) analysis revealed that compared to the ground controls, HU-H_2_O samples exhibited a significant increase in *Clostridium_XIVa*, *Clostridium_IV*, *Blautia*, *Butyricicoccus*, pathogen-containing *Clostridium XIVb* [29], and *Lachnospiraceae_incertae_sedis* and a reduction in *Allobaculum*, *Akkermansia*, *Lactobacillus*, and *Bifidobacterium* (Figure 4C). In comparison to HU-H_2_O samples, HU-Rb1 samples showed higher levels of Allobaculum, *Akkermansia*, *Lactobacillus*, and Barnesiella (Figure 4C). An increase in *Lactobacillus* was also observed in Rb1-treated control mice compared to H_2_O-treated control mice (Figure 4C). These results indicate that Rb1 treatment alleviated HU-induced intestinal dysbiosis, leading to increased levels of probiotics that have been reported to play a beneficial role in ISC homeostasis.

### 2.5. Rb1 Protected HU-Induced ISC Injury via Microbiota and/or Microbiota-Associated Metabolites

**Figure 5:**
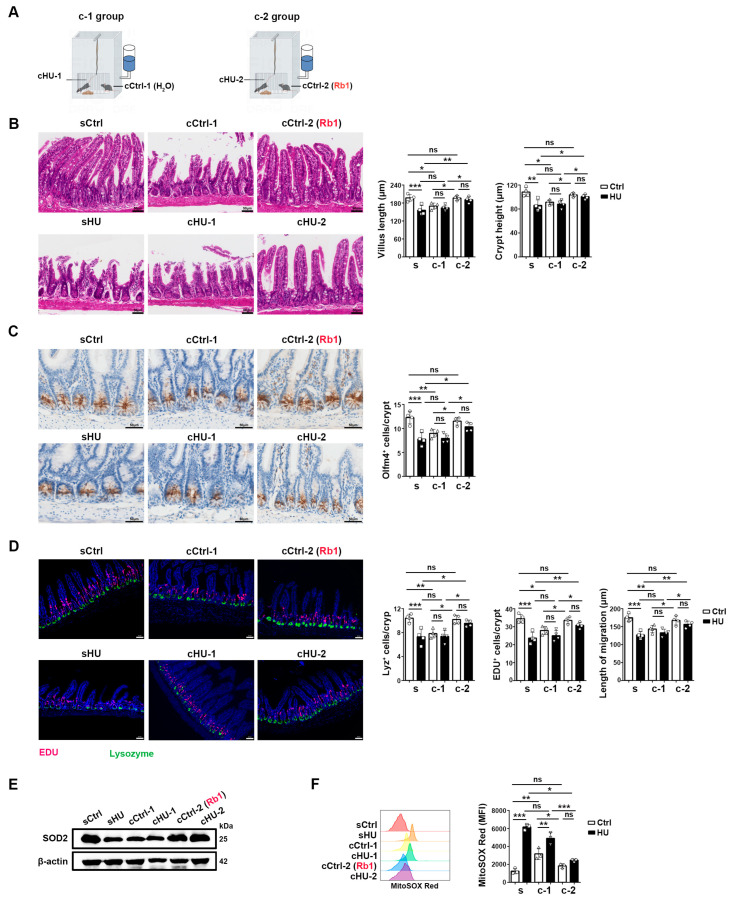
Rb1 protected HU-induced ISC injury via microbiota and/or microbiota-associated metabolites. (**A**) Schematic demonstration of the co-housing experimental setting. C57BL/6 mice were divided into three groups: s group consisting of sCtrl, single-housed ground control mice, and sHU, single-housed HU mice; c-1 group consisting of cCtrl-1 and cHU-1 that were co-housed together, and cCtrl-1 was given H_2_O every day for 4 weeks; c-2 group consisting of cCtrl-2 (Rb1) and cHU-2 that were co-housed together, and cCtrl-2 (Rb1) was given Rb1 at 50 mg/kg by oral gavage every day for 4 weeks. *n* = 4. (**B**) H&E staining of the ileum epithelium. The scale bar is 50 μm. The representative sections are shown on the left. Villus length and crypt depth are summarized on the right. (**C**) Comparison of immunostaining of ileum sections with Olfm4 antibody. The representative sections are shown on the left. The scale bar is 50 μm. The numbers of Olfm4-positive cells per crypt are summarized on the right. In total, 20–30 crypts were counted. (**D**) Comparison of immunostaining of ileum sections with Lysozyme (Lyz, green) and EDU (red) antibodies. The representative sections are shown on the left. The scale bar is 50 μm. The numbers of Lysozyme-positive cells per crypt, EDU^+^ cells per crypt, and the migration distance of EDU^+^ cells are summarized on the right. In total, 20–30 crypts were counted. (**E**) Western blotting of the expression of SOD2 in the ileum epithelium. (**F**) Flow cytometry analysis of mitochondrial reactive oxygen species (mROS) by MitoSOX Red. Representative plots are shown. The mean fluorescence intensities (MFIs) of mROS are summarized on the right. Data are shown as mean ± SD. * *p* < 0.05, ** *p* < 0.01, *** *p* < 0.005, ns, non-significant by unpaired two-tailed Student’s *t*-test.

To investigate whether Rb1 improves ISC homeostasis directly or indirectly through the microbiota, we administered Rb1 to only the ground control mice (cCtrl-2 (Rb1)) that were co-housed with HU mice (cHU-2) for a duration of 4 weeks (Figure 5A). Consistent with our previous report [15], co-housing with HU mice (cHU-1) resulted in intestinal injury in the ground control mice (c-Ctrl-1), as evidenced by reduced villus lengths, crypt heights, Olfm4^+^ ISCs, Lyz^+^ Paneth cells, EDU^+^ proliferating cells, reduced SOD2 level, and increased mitochondrial reactive oxygen species (mROS) level (Figure 5B–F). However, Rb1 treatment in ground control mice (cCtrl-2(Rb1)) prevented intestinal damage both in the co-housed ground control mice (cCtrl-2(Rb1)) and HU mice (cHU-2) (Figure 5B–F). These results suggest that Rb1 protects ISCs and their niche, either through the intestinal microbiota or through microbiota-associated metabolites.

### 2.6. Rb1 Derivative Promoted the Growth of Akkermansia and Bifidobacterium

**Figure 6:**
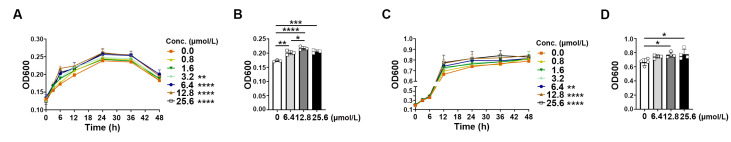
Rb1 derivative CK promoted the growth of *Akkermansia muciniphila* and *Bifidobacterium animalis*. The probiotics were grown in their respective growth media in the presence or absence of CK under anaerobic conditions. OD600 was measured at the indicated time intervals. The mean OD600 values were calculated and compared. (**A**) Comparison of the growth curves of *Akkermansia muciniphila* in the presence and absence of CK. *n* = 3. (**B**) Comparison of the growth of *Akkermansia muciniphila* at 6 h after CK treatment. *n* = 3. (**C**) Comparison of the growth curves of *Bifidobacterium animalis* in the presence and absence of CK. *n* = 4. (**D**) Comparison of the growth of *Bifidobacterium animalis* at 12 h after CK treatment. *n* = 4. Data in (**A**,**C**) are shown as mean values. A two-way ANOVA was used for statistical analysis between the groups with CK and the ones without (0 μM). The data in (**B**,**D**) are shown as mean ± SD. * *p* < 0.05, ** *p* < 0.01, *** *p* < 0.005, **** *p* < 0.001 by ordinary one-way ANOVA.

We further used a bacterial in vitro culture system to examine whether Rb1 has a direct impact on probiotics. As intestinal bacterial strains with glycoside hydrolysis capacity have been shown to transform Rb1 into compound K (CK) to increase the bioavailability and pharmacological benefits of Rb1 [30], we also tested the effect of CK on probiotic growth. As shown in Figure 6, CK significantly promoted the expansion of *Akkermansia muciniphila* and *Bifidobacterium animalis* at 6.4, 12.8, and 25.6 μM. We did not observe a growth difference in Rb1-treated bacteria, likely due to the fast growth of the bacteria and the low bioavailability of Rb1 relative to CK. These results indicate that the Rb1 derivative is capable of promoting probiotic growth in vitro.

## 3. Discussion

ISCs and their continuous renewal of intestinal epithelial cells are crucial for adapting to the challenges posed by microgravity. This study revealed that a simulated weightlessness model of hindlimb unloading resulted in defective homeostasis of ISCs and epithelial cells. The dysregulated composition of the intestinal microflora, reduced Paneth cells, elevated FoxO signaling, and impaired Wnt signaling were associated with the decline in Olfm4^+^ ISCs in HU mice. The supplementation with ginsenoside Rb1 in HU mice, as well as in co-housed ground control mice, alleviated the intestinal damage observed in HU mice. 

The altered composition of the intestinal microflora has been reported in both space-flown mice and ground-based hindlimb unloading models. While the specific bacterial species affected may differ due to variations in detection methods, our study, along with the study by Zhao et al., observed a downregulation of *Akkermansia* and *Lactobacillus* and an upregulation of *Lachnospiraceae* in HU mice [20,21], suggesting a common feature of dysbiosis in the HU mouse model. The exact reasons for the microbiota changes in HU mice remain unclear, but our co-housing experiments and previous fecal transplantation experiments [14] demonstrate that dysbiosis significantly contributes to epithelial injury and ISC dysfunction in HU mice.

Ginseng, revered as the “king of herbs”, possesses various biological activities. The components of ginseng, such as ginsenoside Rb1, have been previously shown to promote intestinal epithelial wound healing and improve epithelial aging. Our data suggest that Rb1 could also enhance Wnt signaling and promote Paneth cell survival while suppressing the FoxO signaling pathway, thereby enhancing ISC homeostasis and differentiation in HU mice. Additionally, treatment of ground control mice with Rb1 alleviated ISC and epithelial cell injury in co-housed HU mice, suggesting that the ISC-promoting function of Rb1 requires the presence of intestinal microbiota. 

Rb1 treatment has been shown to modulate the gut microbiota in a variety of disease models [26,31,32,33,34,35,36,37]. In the ileum of Rb1-treated HU mice, we also found increased levels of *Lactobacillus*, *Akkermansia*, and *Bifidobacterium*. We further showed in an in vitro bacteria culture system that the Rb1 derivative, CK, directly promoted the growth of *Akkermansia* and *Bifidobacterium*. Thus, Rb1 likely improves the functions of ISCs in vivo via a direct probiotic growth promotion mechanism. It remains unclear how CK selectively regulates the growth of beneficial bacteria. The findings that saponin (CK is a typical kind of saponin) promotes the growth of *Bifidobacterium animalis* and *Lactobacillus casei* by upregulating several bacterial key genes (such as *gatC*, *rpmH*, *ruvA*, et al.) involved in biogenesis and metabolic pathways may establish the basis for further investigation [38]. 

Multiple species/strains of probiotics have been reported to improve intestinal epithelial homeostasis and, likely, ISC functions. For instance, *Lactobacillus* is known to produce essential vitamins and short-chain fatty acids (SCFAs) that are beneficial for the growth and metabolism of epithelial cells [39]. The *Lactobacillus*-derived metabolite indole-3-aldehyde has been shown to accelerate epithelial cell proliferation through the promotion of IL-22 production by lamina propria lymphocytes [40]. Both *Akkermansia muciniphila* and *Bifidobacterium* have been shown to have multiple beneficial effects, including improvement of tight junctions and gut permeability, reduction of inflammation, boosting adaptive immune responses, and protection against pathogenic agents, all of which contribute to intestinal epithelial homeostasis [41,42]. 

Taken together, our study demonstrated that hindlimb unloading as a spaceflight analog leads to a reduced number of ISCs, impaired epithelial proliferation, and compromised barrier functions. Rb1 treatment in HU mice alleviated ISC defects by upregulating Wnt signaling and increasing the number of lysozyme^+^ Paneth cells. We further provided evidence that the effect of Rb1 on intestinal epithelial cells in HU mice primarily relies on its probiotic-promoting function mediated through the intestinal microbiota. Of note, long-term space missions require strict safety measures for medications. Rb1 has been widely studied in a variety of tissues in different disease models, and no severe adverse effects have been reported [43]. In the current 4-week hindlimb unloading experiments, we also did not observe any negative impact of Rb1 treatment on body weight and activity. However, these experiments were not designed to test the potential adverse effects of Rb1. Thus, more microgravity-related research to investigate Rb1’s systemic and local effects beyond the intestine is warranted [43].

## 4. Materials and Methods

### 4.1. Humane Care of Animals

The experimental procedures on the use and care of animals had been approved by the ethics committee of the Peking University Health Science Center. Female C57BL/6 mice at 8–10 weeks old were purchased from Vital River Lab Animal Technology Company (Beijing, China) and maintained at Peking University Health Science Center. The mice were housed in standardized, specific pathogen-free conditions with temperatures maintained at 22–23 °C on a 12:12 h light–dark cycle. All animals were allowed to have standard lab chow and water ad libitum. Acclimatization for a minimum of 3 days to the laboratory conditions was performed before experimental inclusion. 

### 4.2. Hindlimb Unloading and Rb1 Treatment

The mice were randomly allocated to four groups: ground control mice receiving H_2_O (Ctrl-H_2_O), HU mice receiving H_2_O (HU-H_2_O), control mice treated with Rb1 (Ctrl-Rb1), and HU mice treated with Rb1 (HU-Rb1). The HU mice were suspended by their tails at a 30° head-down tilt with no load bearing on the hindlimbs, as described previously [44]. Food and water were made accessible through the use of water bottles, gel packs, and food distributed around the cage floor. The animals demonstrated no adverse effects or pronounced weight loss. Groups of 4–6 mice per treatment per experiment were used due to hindlimb suspension cage limitations. The mice in the HU groups were hindlimb unloaded for 28 days. 

Ginsenoside Rb1 was dissolved in sterile water (50 mg/kg) and administered to Ctrl-Rb1 and HU-Rb1 by oral gavage every day, starting 3 days before the initiation of hindlimb unloading. Ctrl-H_2_O and HU-H_2_O received the same amount of H_2_O by oral gavage.

### 4.3. Co-Housing Experimental Design 

The mice were randomly divided into three groups: the s group consisting of sCtrl, single-housed ground control mice, and sHU, single-housed HU mice (4-week HU); the c-1 group consisting of cCtrl-1 and cHU-1 that were co-housed in the same cages at 1:1 ratio, where cCtrl-1 was given H_2_O by oral garage every day for 4 weeks; and the c-2 group consisting of cCtrl-2 (Rb1) and cHU-2 that were co-housed in the same cages at 1:1 ratio, where cCtrl-2 (Rb1) was given Rb1 at 50 mg/kg by oral gavage every day for 4 weeks. 

### 4.4. Histology and Immunohistochemistry

The distal ileum sections of the small intestine were cleaned and opened longitudinally to expose the lumen. The tissue was then pinned to the silicone plate with the villi side up, fixed in 4% PFA, dehydrated, and embedded in paraffin. Serial sections (4 μm) were prepared and stained with hematoxylin and eosin (H&E). The villus lengths and crypt heights were counted using ImageJ (Version 1.53e). For immunostaining, sections were antigen retrieved by heating for 10 min in 10 mM citric acid (pH 6.0) or 1 mM Tris/EDTA (pH 9.0), blocked with normal goat serum (BOSTER, Wuhan, China) at room temperature for 1 h, and subsequently incubated with the following primary antibodies: Ki67 (Abcam, Cambridge, MA, USA), Olfm4 (CST, Danvers, MA, USA), and 4-HNE (BIOSS, Beijing, China) at 4 °C for 12–16 h. Goat anti-rabbit secondary antibody incubation, SABC incubation, DAB staining, hematoxylin counterstaining, dehydration, clearing, mounting, and microscopic imaging were performed following standard immunohistochemical protocols. Immunofluorescence detection involved similar steps with the use of an anti-lysozyme antibody (Abcam, Cambridge, MA, USA), fluorescently labeled secondary antibodies, and nuclear staining with Hoechst. For EDU tracing experiments, 100 μL of EDU (10 mg/kg, Sigma-Aldrich, St. Louis, MO, USA) dissolved in sterile PBS was injected intraperitoneally 12 h before mouse euthanasia. EDU was visualized using the BeyoClick™ EdU Cell Proliferation Kit with Alexa Fluor 555 (Beyotime, Shanghai, China) according to the manufacturer’s instructions. 

### 4.5. Enrichment of Small Intestinal Epithelial Cells

A 15 cm segment of the distal small intestine was dissected and thoroughly cleaned of the mesenterium, adipose tissue, intestinal contents, and Peyer’s patches. The intestine was then opened longitudinally and cut into 1–2 cm sections. These samples were digested in a 50 mL tube with 20 mL of HBSS (without Ca^2+^ and Mg^2+^), supplemented with 5 mM EDTA, 10 mM HEPES, and 10% FBS. The digestion mixture was incubated for 20 min at 37 °C in a shaking incubator operating at 220 revolutions per minute (rpm). After incubation, the supernatant was filtered through a 100 μm cell strainer and transferred to a fresh 50 mL centrifuge tube. The digestion process was repeated for an additional 20 min. Cells were washed with PBS before further analysis.

### 4.6. ELISA

The concentrations of LBP in plasma were determined using Mouse LBP ELISA Kits (Hycult Biotech, Uden, The Netherlands) according to the manufacturer’s instructions. Concentrations of D-Lactate (D-LAC) in plasma were determined using Mouse D-LAC ELISA Kits (Beijing gersion Bio-Technology Co., Ltd., Beijing, China) according to the manufacturer’s instructions.

### 4.7. Flow Cytometry

Intestinal epithelial cells were stained with LIVE/DEAD Fixable Aqua (Invitrogen, Carlsbad, CA, USA) for 15 min at 4 °C to exclude dead cells. This was followed by a 20 min incubation at 4 °C using the antibodies, including anti-EpCAM-PE-Cy7 (Biolegend, San Diego, CA, USA) and anti-CD45-BV421 (Biolegend, San Diego, CA, USA). After washing, the cells were then stained with MitoSOX Red (Invitrogen, Carlsbad, CA, USA) for 10 min at 37 °C. 

### 4.8. RNA Isolation and Real-Time PCR

Total RNA was extracted from epithelial cells using Trizol (Life technologies corporation, Gaithersburg, MD, USA). The RNA samples (2 μg) were reverse transcribed into cDNAs using the FastQuant RT kit (TIANGEN, Beijing, China) according to the manufacturer’s instructions. Quantitative real-time PCR was performed using FastStart Universal SYBR Green Master (Roche, Basel, Switzerland) on a PCRmax Eco 48 real-time PCR system (Illumina, San Diego, CA, USA), with each sample in triplicate. The sequences of primers include (5′–3′): *Actb*, F, GGCTGTATTCCCCTCCATCG, R, CCAGTTGGTAACAATGCCATGT; *Tjp1* (*ZO1*): F, ACTCCCACTTCCCCAAAAAC, R, CCACAGCTGAAGGACTCACA; *Ocln* (*Occludin*): F, GTCCGTGAGGCCTTTTGA, R, GGTGCATAATGATTGGGTTTG; *Lgr5*, F, CCTACTCGAAGACTTACCCAGT, R, GCATTGGGGTGAATGATAGCA; *Olfm4*, F, AAAGTGACCTTGTGCCTGCC, R, AGGGTTCTCTCTGGATGCTGA. The PCRs were performed with an initial denaturation at 95 °C for 10 min, followed by 40 cycles of 95 °C for 10 s, 60 °C for 30 s, and 72 °C for 15 s. The quantification was based on ∆∆Ct calculations and was normalized to *Actb* as loading controls. The ∆∆Ct value of each HU mouse was calculated relative to the mean ∆Ct value of Ctrl mice at the same time point.

### 4.9. RNA Sequencing and Analysis

Total RNA was extracted from Trizol (Life technologies corporation, Gaithersburg, MD, USA), followed by quality control (QC) of the samples and the selection of appropriate testing methods. mRNA was then isolated, fragmented, and converted into double-stranded cDNA through first- and second-strand synthesis. Following end repair, A-tailing, and adaptor ligation, the cDNA fragments were amplified via PCR. The resulting library underwent further QC based on product requirements. The single-stranded PCR products were circularized, and uncyclized linear DNA was digested. The circularized DNA was replicated into DNA nanoballs (DNBs) and loaded onto patterned nanoarrays for sequencing using the combinatorial Probe-Anchor Synthesis (cPAS) method. Gene set enrichment analysis (GSEA) was performed (http://www.broadinstitute.org/gsea/index.jsp, accessed on 6 April 2020) on log2 expression data of epithelial samples. Gene sets comprise cellular, metabolic, and disease pathways from KEGG, Biocarta, and Reactome derived from the Molecular Signatures Database (http://www.broadinstitute.org/gsea/msigdb/index.jsp, accessed on 6 April 2020). Signatures were considered significantly enriched at FDR ≤ 25% when using default parameters and 1000 permutations of gene sets. The RNA sequencing data generated in this study are available at Gene Expression Omnibus GSE89277.

### 4.10. Western Blotting

Total protein extraction was conducted using RIPA lysis buffer. The lysates were mixed with SDS loading buffer and resolved via SDS-PAGE, subsequently transferred onto NC membranes (Millipore, Bedford, MA, USA). The membranes were then blocked with 5% nonfat milk and incubated with primary antibodies against β-actin (Proteintech, Wuhan, China), ZO-1 (Abcam, Cambridge, MA, USA), Occludin (Abcam, Cambridge, MA, USA), and SOD2 (CST, Danvers, MA, USA). After thorough washing in TBST, the membranes were further incubated with the appropriate secondary antibody. Finally, the protein blots were visualized using an ECL detection kit (Tanon, Shanghai, China).

### 4.11. 16s rRNA Sequencing and Data Analysis

The ileum contents were collected into separate autoclaved 1.5 mL centrifuge tubes and labeled accordingly. The 96-well, deep-well plates were prepared with buffers and reagents for DNA extraction. Samples (100–200 mg) were ground with beads in ATL/PVP-10 buffer, incubated at 65 °C for 20 min, centrifuged, and processed with PCI buffer. The supernatant was then transferred to a magnetic bead-containing plate. Using the Kingfisher system, the DNA extraction process was automated. Extracted DNA underwent quantification, integrity assessment, and library construction. A PCR reaction was set up with 30 ng of DNA and fusion primers, followed by amplification, purification using magnetic beads, and quality control using the Agilent 2100 Bioanalyzer (Agilent, Santa Clara, CA, USA). Qualified libraries were sequenced on the Illumina platform. The resulting data were filtered, assembled into Tags, clustered into OTUs, annotated, and analyzed for species complexity and differences. Cluster analysis was preceded by principal co-ordinates analysis (PCoA), which was applied to reduce the dimension of the original variables using the FactoMine R package and the ggplot2 package. Linear effect size (LEfSe) analysis was performed using the default parameters to identify features that discriminated between the two groups. 

### 4.12. Bacterial Growth and Measurements

*Akkermansia muciniphila* and *Bifidobacterium animalis* strains were maintained in their respective anaerobic conditions. Prior to experimentation, the bacteria were revived by subculturing in anaerobic chambers or bags. Ginsenoside CK (purity > 98%, CAS No. 39262-14-1) was dissolved in sterile dimethyl sulfoxide (DMSO) to prepare the stock solution and further diluted in growth media to achieve the desired concentrations. The final concentration of DMSO in the culture medium did not exceed 0.1% (*v*/*v*), which was determined to have no significant effect on bacterial growth. For each experiment, the bacteria were grown in their respective growth media (MRS broth for *Bifidobacterium animalis* and Brain Heart Infusion for *Akkermansia muciniphila*) supplemented with the indicated concentrations of CK in triplicate or quadruplicate. The cultures were incubated in anaerobic conditions at 37 °C for 48 h.

The optical density (OD) of bacterial culture at 600 nm (OD600) was measured using a microcoder (BioTek, Winooski, VT, USA) to estimate bacterial growth. At the specified time intervals, OD600 values were determined. 

### 4.13. Statistics

The data are presented as mean values of 3–6 mice in each group ± standard deviation (SD). The statistical analysis of the results was performed using GraphPad Prism 6.0 software (San Diego, CA, USA). Statistical significance between the two groups was evaluated by the Mann–Whitney test or two-tailed unpaired Student’s *t*-test. Throughout the text, figures, and figure legends, the following terminology is used to denote statistical significance: * *p* < 0.05, ** *p* < 0.01, *** *p* < 0.001, **** *p* < 0.0001.

## Data Availability

The data presented in this study are available on request from the corresponding author. The data are not publicly available due to institutional data restriction.
